# Risk of arboviral transmission and insecticide resistance status of *Aedes* mosquitoes during a yellow fever outbreak in Ghana

**DOI:** 10.1186/s12879-024-09643-z

**Published:** 2024-07-25

**Authors:** Margaret Owusu-Akyaw, Christopher Mfum Owusu-Asenso, Anisa Abdulai, Abdul Rahim Mohammed, Isaac Kwame Sraku, Emmanuel Nana Boadu, Evans Aduhene, Simon Kwaku Attah, Yaw Asare Afrane

**Affiliations:** Centre for Vector-Borne Disease Research, Department of Medical Microbiology, Medical School, University of Ghana, Accra, Ghana

**Keywords:** *Aedes aegypti*, Yellow fever, Stegomyia indices, Ghana

## Abstract

**Background:**

In late 2021, Ghana was hit by a Yellow Fever outbreak that started in two districts in the Savannah region and spread to several other Districts in three regions. Yellow fever is endemic in Ghana. However, there is currently no structured vector control programme for *Aedes* the arboviral vector in Ghana. Knowledge of *Aedes* bionomics and insecticide susceptibility status is important to control the vectors. This study therefore sought to determine *Aedes* vector bionomics and their insecticide resistance status during a yellow fever outbreak.

**Methods:**

The study was performed in two yellow fever outbreak sites (Wenchi, Larabanga) and two non-outbreak sites (Kpalsogu, Pagaza) in Ghana. Immature *Aedes* mosquitoes were sampled from water-holding containers in and around human habitations. The risk of disease transmission was determined in each site using stegomyia indices. Adult *Aedes* mosquitoes were sampled using Biogents Sentinel (BG) traps, Human Landing Catch (HLC), and Prokopack (PPK) aspirators. Phenotypic resistance to permethrin, deltamethrin and pirimiphos-methyl was determined with WHO susceptibility tests using *Aedes* mosquitoes collected as larvae and reared into adults. Knockdown resistance (kdr) mutations were detected using allele-specific multiplex PCR.

**Results:**

Among the 2,664 immature *Aedes* sampled, more than 60% were found in car tyres. Larabanga, an outbreak site, was classified as a high-risk zone for the Yellow Fever outbreak (BI: 84%, CI: 26.4%). Out of 1,507 adult *Aedes* mosquitoes collected, *Aedes aegypti* was the predominant vector species (92%). A significantly high abundance of *Aedes* mosquitoes was observed during the dry season (61.2%) and outdoors (60.6%) (*P* < 0.001). Moderate to high resistance to deltamethrin was observed in all sites (33.75% to 70%). Moderate resistance to pirimiphos-methyl (65%) was observed in Kpalsogu. *Aedes* mosquitoes from Larabanga were susceptible (98%) to permethrin. The F1534C kdr, V1016I kdr and V410 kdr alleles were present in all the sites with frequencies between (0.05–0.92). The outbreak sites had significantly higher allele frequencies of F1534C and V1016I respectively compared to non-outbreak sites (*P* < 0.001).

**Conclusion:**

This study indicates that *Aedes* mosquitoes in Ghana pose a significant risk to public health. Hence there is a need to continue monitoring these vectors to develop an effective control strategy.

## Introduction

*Aedes* mosquitoes represent an ever-growing threat to public health worldwide due to their ability to transmit many infectious arboviral pathogens such as Dengue, Chikungunya, Zika and Yellow Fever [1]. Yellow fever (YF), an acute viral disease affecting humans and non-human primates (NHP), is caused by the yellow fever virus (YFV) [2]. The virus is transmitted by the bite of infected female *Aedes* mosquitoes [3]. *Aedes aegypti* is one of the vectors for yellow fever in Africa alongside *Ae. albopictus* which is known to be more invasive and also a competent vector for Dengue fever and Chikungunya [4]. The World Health Organisation (WHO) has reported that forty-seven countries in Africa are either endemic or have regions that are endemic for YF and other arboviral infections [5]. Furthermore, the WHO advises countries that have *Aedes* mosquitoes but no evidence of viral transmission to identify local regions with high mosquito densities and make proper preparations to deal with any possible arboviral outbreaks [6]. Yellow fever affects over 200,000 people and causes about 30,000 deaths annually [7]. In Africa, annually an estimated 21 million people are at risk [8].

Yellow Fever is endemic in Ghana; this situation is exacerbated by the limited vaccine coverage, vector abundance and increasing insecticide resistance [9–12], creating a risk for onward transmission and amplification of the virus among unvaccinated populations [10]. Major arboviral disease outbreaks have been recorded in Ghana since 1969 [10]. A recent outbreak of yellow fever was experienced in Ghana in 2021 within the Savannah, Oti, Bono and Upper West regions. Reports from the Ghana Health Service, as of 4th December 2021, 202 suspected cases had been reported with 85 confirmed cases and 46 mortalities [13]. Evidence of the presence and exposure to other arboviral diseases such as dengue fever and chikungunya have also been reported in Ghana [14–16]. These reports show that arboviral pathogens are in circulation in Ghana and require the establishment of effective surveillance and vector control management strategies.

Despite the increasing outbreaks of arboviral diseases and the high densities of the arboviral vectors in Africa, its control is given limited attention [8]. The control of arboviral diseases majorly relies on vector control using insecticides coupled with larval source management and case management. Increasing insecticide resistance in *Aedes* mosquitoes poses a major challenge for vector control strategies. Resistance of the *Aedes* mosquitoes to insecticides has been reported in some West African countries like Senegal, Burkina Faso and Ghana [9, 11, 17, 18]. *Aedes* mosquitoes populations in Ghana have been found to be resistant to several public health insecticides including pyrethroids, organochlorines and carbamates [9, 11, 12, 19]. Target-site mutations such as V410L, V1016I and F1534C have been found in pyrethroid-resistant *Aedes* mosquitoes from Ghana and other countries [9, 20–23].

Vector and insecticide susceptibility surveillance is still crucial in reducing the global burden of arboviral infections [24]. However, there is a paucity of data on the risk of transmission and insecticide susceptibility status of these arboviral disease vectors in Ghana. The current study sought to determine the risk of arboviral transmission and insecticide susceptibility status of the *Aedes* mosquitoes in the selected yellow fever outbreak and non-outbreak areas in Ghana.

## Methods

### Study sites

**Fig. 1:**
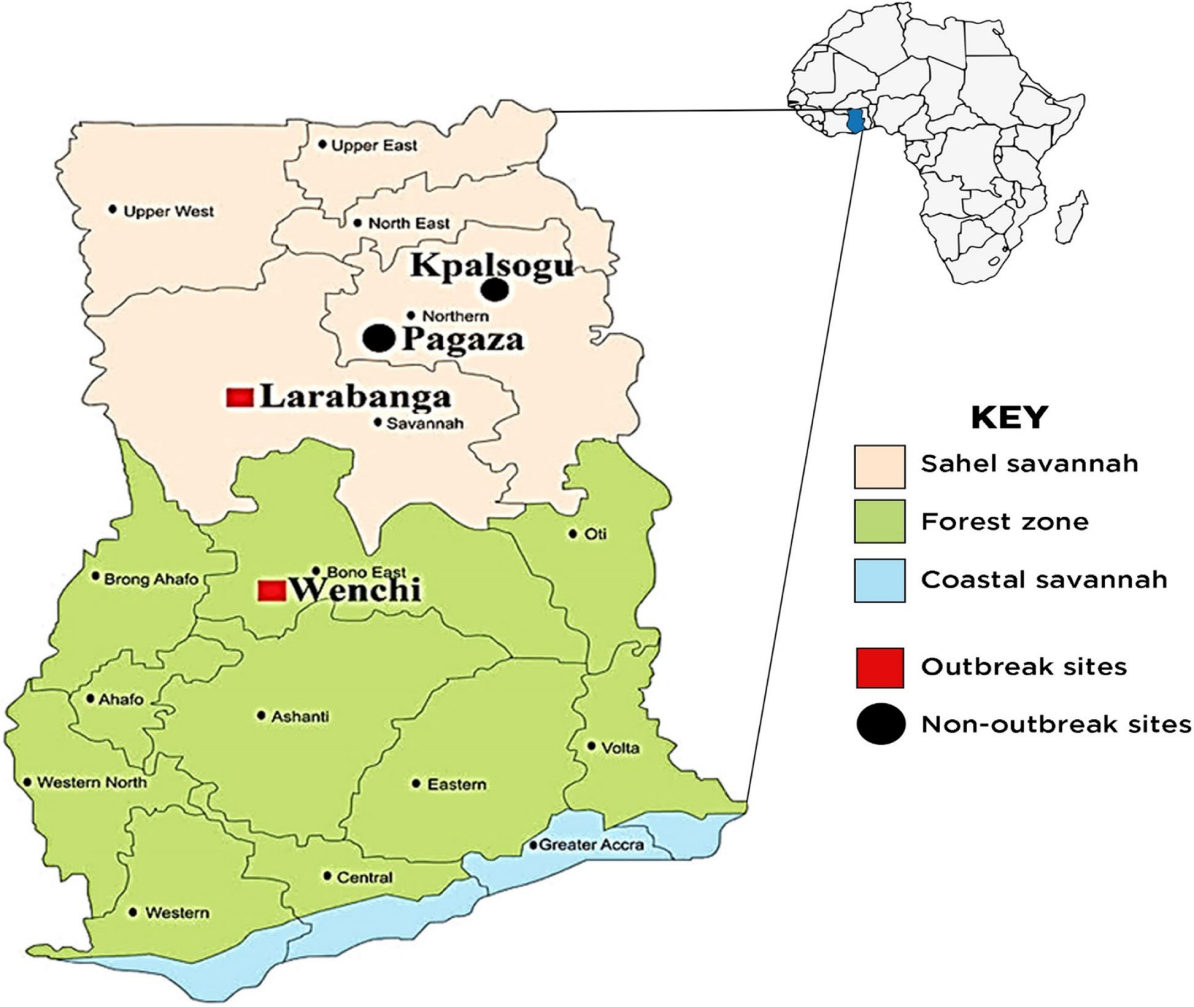
Map of Ghana indicating the study areas

This study was conducted in four sites, two yellow fever outbreak areas [Larabanga (9°5′0″N, 1°49′0″W) and Wenchi (7°33′33″N 1°55′45″W)] and two non-outbreak areas [Kpalsogu (9°33′45.2″N, 1°01′54.6″W) and Pagaza (9°22′33.34″N, 0°42′29.67″W)] as control areas. Of the four sites, three were rural areas (Larabanga, Kpalsogu and Pagaza) located in the Sahel Savannah zone of Ghana and one urban area (Wenchi) in the forest zone of Ghana (Fig. 1). Sampling of mosquitoes was done during the dry (April – June) and rainy (August – October) seasons. The control sites were selected based on their similar ecologies, which allows for a comparative analysis under similar environmental conditions. The control sites were located over 50 km from the outbreak sites, which exceeds the flight range of *Aedes* mosquitoes [25] thereby minimizing the likelihood of significant mosquito movement between the sites. Water supply in the rural study sites (Larabanga, Kpalsogu and Pagaza) is mainly from harvested rainwater, wells, and boreholes, and in Wenchi, there is irregular piped water supply system. In all the sites, access to pipe-borne water was a big challenge; therefore, households tend to store water in storage containers, pots, and drums for long-term use, which creates breeding sites for *Aedes* mosquitoes.

### Characterization of *Aedes* breeding habitats and abundance of *Aedes* larvae

Larval sampling was performed in each study site, to characterise the breeding habitats and abundance of immature *Aedes* mosquitoes. During the larval surveys, the habitat type, its location in a household (indoor or outdoor), and its physical characteristics were recorded. Six habitat types were classified based on their container types: car tyres, discarded containers, drinking pots, drums, tanks, and buckets using well-described protocols by Owusu-Asenso et al*.* [11]. Discarded containers and drinking pots were 50–100 L capacity containers which included broken jars, bottles, small plastic food containers, tins, plates, cans, cooking pots, drinking troughs and broken pots made of clay, plastic or metal.

**Fig. 2:**
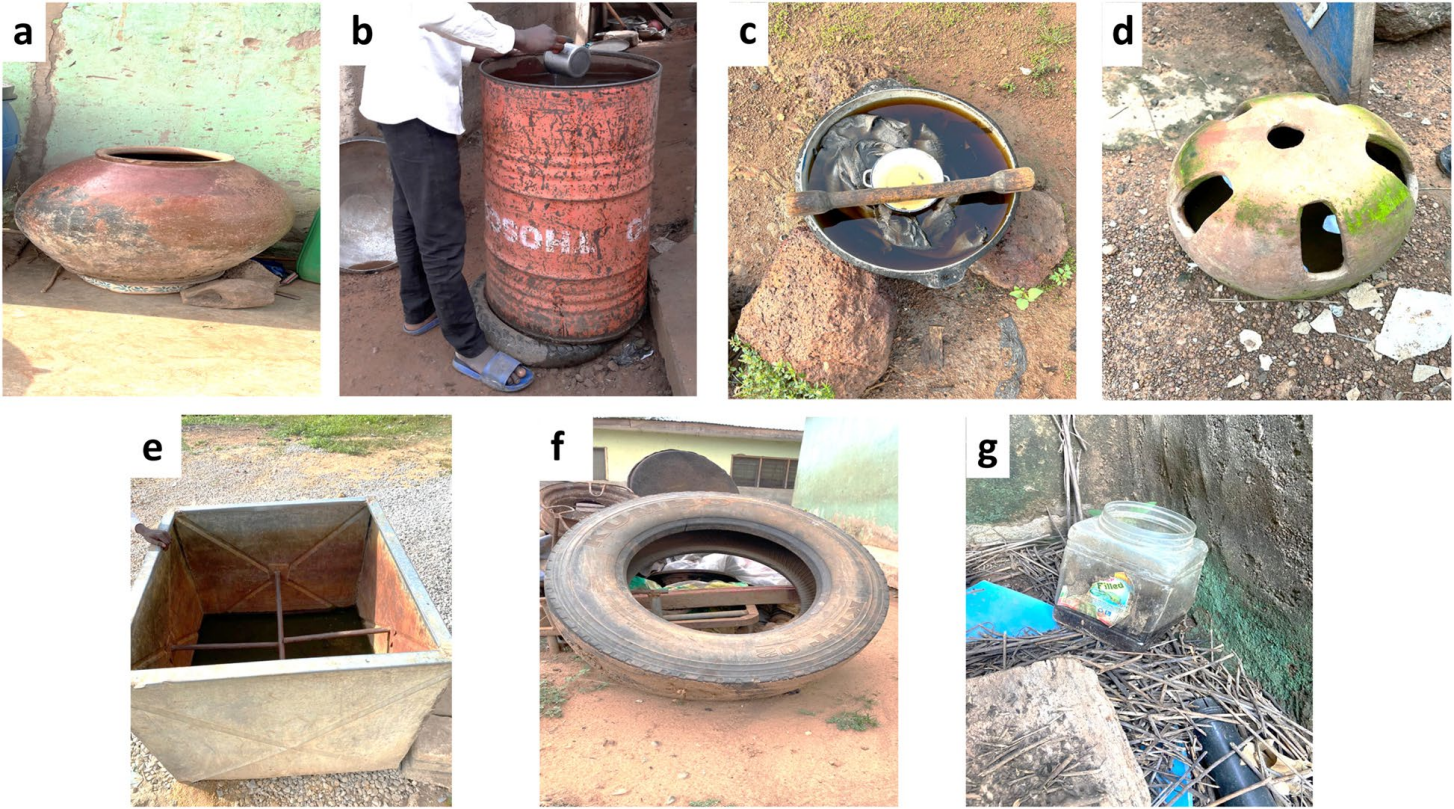
Larval habitats encountered during larval sampling. **a** – **g**. **a** Drinking pot, **b** metal drum, **c** metal pan, **d** drinking trough, **e** metal tank, **f** Car tyre, **g** Discarded container

Drums were defined as 100-500L capacity plastic water storage containers. Tanks were 100–500 L capacity water storage containers made of metal or concrete. Buckets included 10–25 L water storage containers made of metal or plastic (Fig. 2). Coordinates of all collection points were recorded using a GPSMAP® 60CSx geographical position system (GPS) instrument (Garmin International, Inc., Olathe, Kansas, USA). *Aedes* larvae from positive containers were collected using pipettes and ladles using well- described protocols [11]. For larger containers, the water was first sieved and larval samples placed in a white plastic tray with some water. All larval samples were transported to the insectary at the Department of Medical Microbiology, University of Ghana, where they were raised to adults under suitable conditions (temperature: 27 ± 2 °C, 75 ± 10% relative humidity). The larvae were fed on TetraMin Baby fish food (Tetra Werke, Melle, Germany). Emerged adults were morphologically identified using standard taxonomic keys [25].

### Determination of Stegomyia indices

The extent of infestation by *Aedes* mosquitoes was estimated using the classical Stegomyia indices including BI, Breteau Index, (the number of positive containers per 100 surveyed houses); HI; House Index, (percentage of houses positive for *Aedes* larvae or pupae); CI; Container Index, (the percentage of containers positive for *Aedes* larvae or pupae per 100 houses inspected) [26, 27]. These larval and pupal indices continue to be the predominant and frequent measures used to evaluate vector prevalence. This is because catching adult mosquitoes is time-consuming and requires access to private land [12]. Stegomyia indices are quantitative indicators of the risk of transmission.

Using the WHO criteria, the risk of YF at each site was assessed as follows: In an area where BI, HI, and CI exceeded 50%, 35% and 20% respectively, the risk of *Ae aegypti*-transmitted Viral Haemorrhagic Fever (VHF) was considered to be high; an area where BI was between 5 and 50%, the density of *Ae. aegypti* was considered to be sufficient to promote an outbreak of VHF disease; an area where BI, HI and CI were less than 5%, 4% and 3% respectively, it was considered to be unlikely for YF transmission to occur [28].

### Adult *Aedes* mosquito sampling

The spatio-temporal distribution of adult *Aedes* mosquitoes was determined by sampling indoors and outdoors using three methods; Biogent sentinel 2 traps (BG traps), Human landing catches (HLC), and prokopack aspiration (PPK) (John W. Hock Company, Gainesville, U.S.A.) [29]. Geographical coordinates of each house sampled were taken. Cross-sectional surveys were undertaken in the dry season (March to May 2022) and in the rainy season (August to October 2022). Sixteen houses were randomly selected for each sampling method at each site (4 houses per day for four consecutive days). Biogent sentinel traps were set both indoors (living rooms and bedrooms) and outdoors (open, verandas, or under a shed/tree where people sit to chat) about 5 m from the house) during the times 3:00–7:00 pm. The BG traps were baited with carbon dioxide (CO_2_) which was produced from a mixture of 17.5 g yeast (Angel Yeast (Egypt) Co. Ltd.), 250 g sugar in 1 L of water [11]. After the 4 h, mosquitoes trapped were carefully removed, placed in a cooler box containing ice and then transported to the insectary. The HLC method was also used to sample host-seeking adult *Aedes* mosquitoes. On each day, one trained volunteer was positioned indoors and another outdoors and collected mosquitoes from 3:00 -6:00 pm. Collected *Aedes* were placed in well-labelled paper cups, and transported to the insectary for identification and further processing.

Prokopack aspirations were used to sample resting mosquitoes. Sampling for *Aedes* mosquitoes was done indoors and outdoors. Sampled adult *Aedes* mosquitoes were knocked down with chloroform and preserved in Eppendorf tubes containing silica gel.

### Morphological identification of *Aedes* mosquitoes

All adult *Aedes* mosquitoes from the adult sampling, and those used for the susceptibility testing (*Aedes* larvae collected in the study sites and raised to adults), were identified morphologically using the taxonomic keys by Huang [25].

### Insecticide susceptibility tests

Insecticide susceptibility test was conducted using WHO tubes to determine phenotypic resistance according to WHO criteria [30]. Adult female *Aedes* mosquitoes that were 3 – 5-day-old were exposed to papers impregnated with permethrin (0.75%), deltamethrin (0.05%), and pirimiphos-methyl (0.25%). Larvae and pupae collected from the larval sampling as well as the Ovitraps that were set were used for the WHO susceptibility testing. Though these doses are not the recommended doses for evaluating the susceptibility of *Aedes* mosquitoes, they are the most commonly used [11, 31]. These concentrations were used because WHO-recommended concentrations for *Aedes* mosquitoes were not available during the bioassay. The WHO insecticide concentrations are lower than that of *Anopheles* that were used in this study, currently the recommended concentrations are 0.03% for deltamethrin, 0.25% for permethrin, and 0.21% for pirimiphos-methyl [9]. The knockdown time was recorded every 10 min during the 60-min exposure period. Mortalities were recorded after a 24-h recovery period. Alive (resistant) and dead (susceptible) mosquitoes were stored in absolute ethanol for later DNA analysis.

### Genotyping of kdr mutations in *Aedes* aegypti populations

A sub-sample of 242 phenotypically pyrethroid resistant and susceptible *Aedes* mosquitoes were genotyped of kdr mutations, F1534C, V1016I and V410L. Total DNA was extracted from whole mosquitoes using the DNeasy Tissue Kit (Qiagen, In USA). Genotyping of the kdr mutations was done using allele-specific multiplex PCR according to well-described protocols of Villanueva-Segura et al*.* [32].

### Statistical analysis

A descriptive analysis was conducted to assess the variation in larval and adult abundance across the sites, seasons and indoor versus outdoor locations. Chi-square was used to determine significant differences in the mosquito abundance observed. WHO insecticide susceptibility tests were analyzed using the WHO criteria [28]. Mosquitoes were classified as susceptible if the mortality rate was between 98 and 100%; as suspected resistant if the mortality rate was between 90 and 97%; as resistant if the mortality rate was < 90% [28]. Knockdown and mortality rates were compared between sites using Chi-square. Allele frequencies were calculated according to the Hardy-Weinburg Equilibrium formula [33].

$$\text{F}\left(\text{kdr}\right)=\frac{2\text{RR }+\text{ RS}}{2\text{n}},$$

where RR is the number of homozygotes, RS is the number of heterozygotes, and n is the total number of specimens analyzed.

## Results

### Distribution and abundance of *Aedes* larval habitats

A total of 535 larval habitats with 86 positive breeding habitats were surveyed from all the study sites, comprising six [6] different habitat types over the entire sampling period. Overall, the most abundant habitat type was car tyres [59.3%, *n* = 51/86], whereas the least were tanks [2.3%, *n* = 2/86] and buckets [2.3%, *n* = 2/86] (Table 1). A significantly higher abundance of larval habitats was encountered during the rainy season [62.8%, *n* = 54/86] than in the dry season [37.2%, *n* = 32/86] (*X*^2^ = 18.5035, *df* = 5, *P* = 0.002). Within the 4 sites sampled, a significantly higher proportion (99.8%, 85/86) of larval habitats were encountered outdoors as compared to indoors (1.2%, 1/86) (*X*^2^ = 42.4941, *df* = 5, *P* < 0.001). Car tyres were the most predominant habitat type in the outbreak sites, Wenchi [81.8%, *n* = 9/11] and Larabanga [74.1%, *n* = 34/43] and non-outbreak site, Pagaza [43.8%, *n* = 7/16]. However, in non-outbreak site, Kpalsogu, drinking pots [81.3%, *n* = 13/16] (*X*^2^ = 69.72, *df* = 5, *P* < 0.001) (Table 1).

**Table 1:** Positive larval habitat distribution abundance per study site

	Outbreak sites		Non-outbreak sites		
	Wenchi N (%)	Larabanga N (%)	Kpalsogu N (%)	Pagaza N (%)	Total N (%)
**Bucket**	0	1 (2.3)	0	1 (6.2)	2 (2.3)
**Discarded container**	1 (9.1)	1 (2.3)	0	4 (25)	6 (7)
**Drinking pot**	0	3 (7)	13 (81.3)	4 (25)	20 (23.2)
**Drum**	1 (9.1)	2 (4.6)	2 (12.5)	0	5 (5.9)
**Tank**	0	2 (4.6)	0	0	2 (2.3)
**Car Tyre**	9 (81.8)	34 (79.2)	1 (6.2)	7 (43.8)	51 (59.3)
**Total**	**11 (12.8)**	**43 (50)**	**16 (18.6)**	**16 (18.6)**	**86 (100)**

*N* frequency

### Larval abundance of *Aedes* immature

A total of 2,664 *Aedes* immatures were collected over the entire sampling period, of which the most productive habitat type was car tyres 65.1% (1,734/2664), whereas the least productive was tanks 1.1% (30/2664) (Table 2). *Aedes* larval abundance was slightly higher at 1,342 (50.4%) in the rainy season as compared to the dry season at 1,322 (49.6%) (*X*^2^ = 37.1991, *df* = 28, *P* = 0.115). During both seasons, the highest larval abundance was observed in Larabanga, a yellow fever outbreak site [*n* = 1,472 (55.3%)], whereas the least was observed in Wenchi, also an outbreak site [n = 217 (8. 1%)], Table 2. A significantly higher abundance of *Aedes* immatures were collected outdoors [*n* = 2,626 (98.6%)] as compared to [*n* = 38 (1.4%)] those collected indoors (*X*^2^ = 86.000, *df* = 28, *P* < 0.001), (Table 2).

**Table 2:** The seasonal distribution of *Aedes* immatures across the study sites

	**Wenchi**		**Larabanga**		**Kpalsogu**		**Pagaza**	
**Location**	**Dry**	**Rainy**	**Dry**	**Rainy**	**Dry**	**Rainy**	**Dry**	**Rainy**
Indoor	0	0	0	0	0	38	0	0
Outdoor	145	72	832	640	0	392	345	200
**Container Type**								
Bucket	0	0	0	0	0	38	0	42
Discarded container	20	0	0	75	0	122	0	101
Drinking pot	0	0	0	75	0	210	30	57
Drum	10	0	0	75	0	45	0	0
Tank	0	0	15	15	0	0	0	0
Car Tyre	115	72	817	400	0	15	315	0
**Total**	**145**	**72**	**832**	**640**	**0**	**430**	**345**	**200**

### Larval Indices (Stegomyia Indices)

**Fig. 3:**
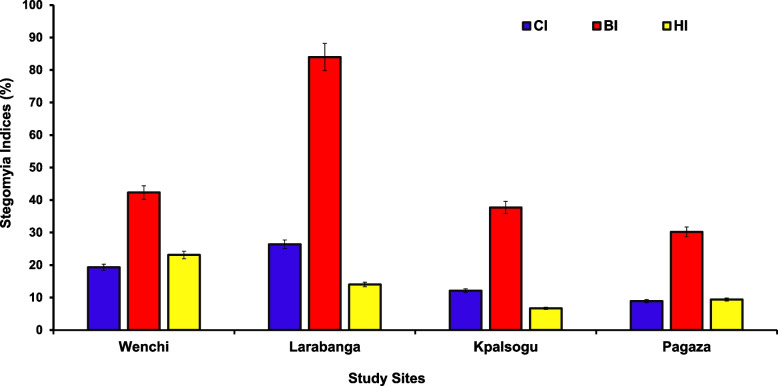
Stegomyia Indices per study site

Larval Indices calculated using the WHO formula for each study site were used to make inferences about the risk of transmission of yellow fever in the outbreak and non-outbreak zones studied. From the results obtained, in Larabanga the indices (BI: 84%, CI:26.4%, HI:14) exceeded the threshold and can be classified as a high-risk zone for yellow fever transmission as compared to all the other study areas, Wenchi (BI:42.3% CI:19.3% HI:23.1%), Kpalsogu (BI:37.7%, CI:12.1%, HI:6.7%) and Pagaza (BI: 30.2%, CI:8.9%, HI:9.4%) that had values within the range sufficient to promote an outbreak. These are shown in Fig. 3.

### Spatio-temporal distribution of adult *Aedes* mosquitoes

Overall, a total of 1,507 adult *Aedes* mosquitoes were collected from all the study areas, with *Aedes aegypt*i [92%, *n* = 1386/1507] as the predominant species, followed by *Aedes formosus* [8%, *n* = 121/1507]. A high abundance of *Aedes* mosquitoes was collected in Larabanga, a yellow fever outbreak site [*Aedes aegypti* 884/1386 (63.8%); *Aedes formosus* 112/121 (92.3%)], whereas the least abundance was collected in Pagaza, a non-yellow fever outbreak site [*Aedes aegypti* 75/1386 (5.4%); *Aedes formosus* 9/121 (7.7%)] (*X*^2^ = 52.061, *df* = 3, *P* < 0.001).

A high abundance of *Aedes* mosquitoes was sampled outdoors [*n* = 914 (60.7%)] as compared to indoor collections [*n* = 593 (39.3%); *X*^2^ = 68.38, *df* = 1, *P* < 0.01)]. A high abundance of adult *Aedes* mosquitoes was sampled for both indoor [*n* = 342 (34.3%)] and outdoor [*n* = 654 (65.7%)] collections in Larabanga whereas, the least recorded abundance was in Pagaza [Indoor (*n* = 45 (53.6%); Outdoor (*n* = 39, 46.4%; *X*^2^ = 32.021, *df* = 3, *P* < 0.001), Table 3.

**Table 3:** Adult *Aedes* abundance per study site and location

Study site	*Aedes aegypti* N (%)		*Aedes formosus* N (%)		Total per Site N (%)
	**Indoor**	**Outdoor**	**Indoor**	**Outdoor**	
**Wenchi**	120 (36.9)	205 (63.1)	0 (0.0)	0 (0.0)	325 (21.5)
**Larabanga**	110 (12.4)	774 (87.6)	0 (0.0)	112 (12.0)	996 (66.6)
**Kpalsogu**	0 (0.0)	102 (100)	0 (0.0)	0 (0.0)	102 (6.7)
**Pagaza**	5 (6.7)	70 (93.3)	0 (0.0)	9 (11.0)	84 (5.6)
**Total**	**235 (17.0)**	**1151 (83.0)**	0 (0.0)	**121 (8.0)**	**1507 (100)**

***N*** Sample size

**Fig. 4:**
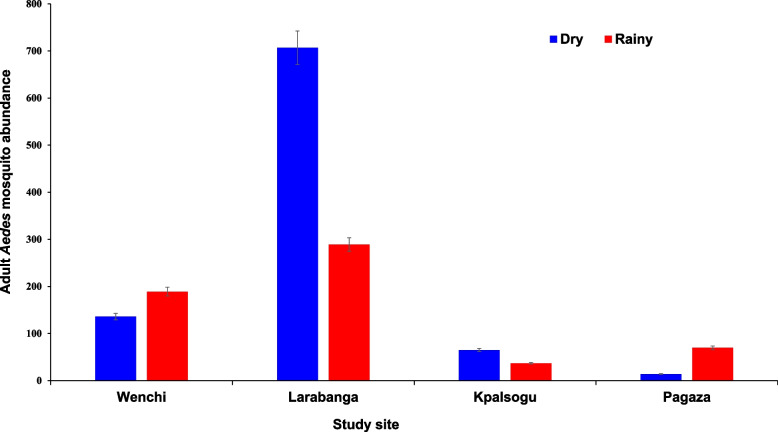
Seasonal abundance of adult *Aedes* mosquitoes

Adult *Aedes* mosquitoes were more predominant in the dry season [*n* = 922/1507 (61.2%)] than in the rainy season [*n* = 585/1507 (38.8%)], (*X*^2^ = 75.36, *df* = 1, *P* < 0.001). In the dry season, the highest abundance of adult *Aedes* mosquitoes was recorded in Larabanga [*n* = 289/585, (49.4%)], followed by those from Wenchi [*n* = 189/585, (32.3%)], both being yellow fever outbreak sites. Mosquitoes from the non-yellow fever outbreak sites were the least, Pagaza [*n* = 70/585, (12.0%)] and Kpalsogu [*n* = 37/585, (6.3%)]. Similarly, during the rainy season, the highest abundance of *Aedes* mosquitoes was recorded in Larabanga [*n* = 707/922, (76.7%)], followed by Wenchi [*n* = 136/922 (14.8%)], the yellow fever outbreak sites. There were small numbers of *Aedes* mosquitoes sampled in the non-yellow fever outbreak sites of Kpalsogu [*n* = 65/922, (7.0%)] and Pagaza [*n* = 14/922, (1.5%)]. These are shown in Fig. 4.

### Seasonal abundance of adult *Aedes* mosquitoes per trap type

**Fig. 5:**
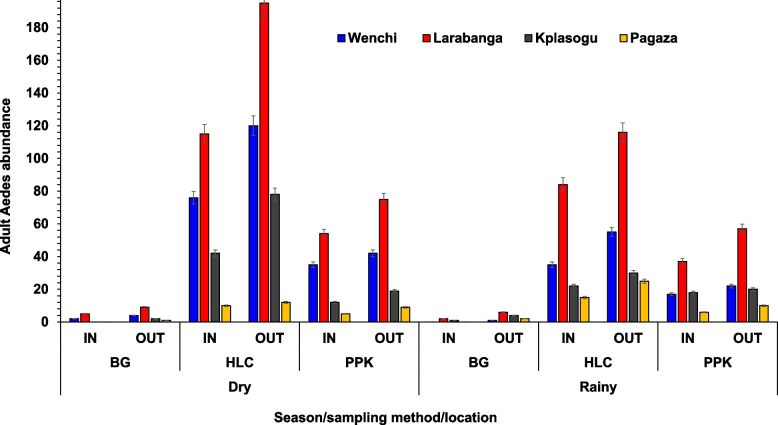
Season abundance of *Aedes* mosquitoes per trap type (In-Indoor; OUT-Outdoor; BG-Biogents-2 sentinel traps; HLC-Human Landing Catches; PPK-Prokopack aspiration)

Three separate traps namely, HLC, PPK, and BG were used to collect a total of 1507 adult *Aedes* mosquitoes from all the study sites. A total of 601 (39.9%) *Aedes* mosquitoes were collected using HLC [dry = 280 (46.6%); rainy = 321 (53.4%)], PPK 443 (29.4%) [dry = 183 (41.3%); rainy = 260 (58.7%)] and BG 463 (30.7%) [dry = 122 (26.3%); rainy = 341 (73.7%)]. Of the 796 female adult mosquitoes caught with PPK and BG, 575 [PPK = 295 (66.6%); BG = 280 (60.5%)] were unfed, 177 [PPK = 77 (17.4%) BG = 100 (21.6%)] were blood-fed, half-gravid [PPK = 12 (2.7%); BG = 0] and gravid [PPK = 18 (4.1%); BG = 14 (3.0%)], (*X*^2^ = 46.745, *df* = 2, *P* < 0.001) (Fig. 5).

### Insecticide susceptibility status of *Aedes* mosquitoes

**Fig. 6:**
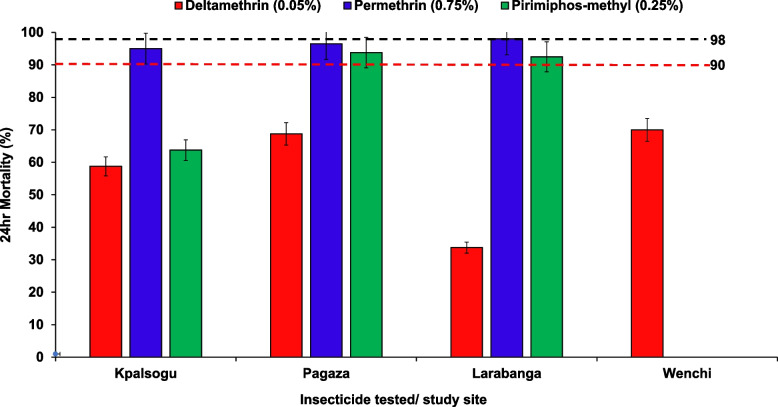
Mortalities of *Aedes* mosquitoes exposed to different insecticides in the study sites

Bioassay results showed resistance to deltamethrin at × 1 concentration across all the study sites (33.75%—70%) (Fig. 6). The mosquitoes showed possible resistance to permethrin at × 1 in Kpalsogu (95%), Pagaza (96.5%), and susceptibility in Larabanga (98%). Resistance to pirimiphos-methyl at × 1 was shown in *Aedes* mosquitoes in Kpalsogu (63.75%) and possible resistance was observed in Pagaza (93.75%) and Larabanga (92.5%). For Wenchi, for logistical challenges, not many larvae were sampled for the determination of insecticide susceptibility, so only deltamethrin WHO susceptibility tests were performed. These results are shown in Fig. 6.

### Genotypic mutations associated with resistance in *Aedes* aegypti

A subset of 242 *Ae. aegypti* obtained from the phenotypic assays were genotyped for the F1534C, V1016I and V410L kdr mutations. About 20–30 mosquito samples were selected from each site per insecticide paper per concentration for the genotypic resistance determination. Permethrin exposed mosquito samples from Kpalsogu and Wenchi were not genotyped because of logistic challenges. The F1534C mutation was detected in moderate to high allele frequencies in *Ae. aegypti* mosquitoes exposed to pyrethroid insecticides. *Aedes aegypti* from Wenchi that were exposed to deltamethrin, had a significantly high allele frequency of F1534C mutation (0.92) compared to mosquitoes from the non-outbreak sites, Pagaza (0.19) and Kpalsogu (0.35) (*χ2* = 50.50, *df* = 3, *P* < 0.001). For the V1016I mutation, low to moderate allele frequencies (0.23 to 0.54) were observed in *Ae. aegypti* mosquitoes were exposed to both deltamethrin and permethrin except for Larabanga, an outbreak site, where mosquitoes had a high allele frequency of 0.77. However, there was a significant difference in the frequency of V1016I mutation in outbreak and non-outbreak sites (*P* < 0.05). For the V410L mutation, there was no significant differences in the frequency of the mutations between outbreak sites and non-outbreak sites with low allele frequencies ranging from 0.05 to 0.15. The genotypes and allele frequencies of each kdr mutation are shown in Table 4.

**Table 4:** Number of genotypes and frequencies of the F1534C, V1016I and V410L mutation in the voltage-gated sodium channel gene of *Aedes aegypti* mosquitoes

				**F1534C**				**V1016I**				**V410L**			
**Insecticide**	**Site Description**	**Study site**	**n**	**CC**	**FF**	**FC**	**Allele** **Freq**	**II**	**VV**	**VI**	**Allele** **Freq**	**LL**	**VV**	**VL**	**Allele** **Freq**
**Deltamethrin**	**Non-outbreak**	**Pagaza**	60	6	43	11	0.19	3	22	35	0.34	0	45	15	0.13
		**Kpalsogu**	44	12	25	7	0.35	1	1	42	0.5	3	40	1	0.08
	**Outbreak**	**Larabanga**	30	8	13	9	0.42	2	0	28	0.53	0	27	3	0.05
		**Wenchi**	52	45	1	6	0.92	8	4	40	0.54	3	46	3	0.09
		**Total**	**186**	**71**	**82**	**33**		**14**	**27**	**145**		**6**	**160**	**22**	
**Permethrin**	**Non-outbreak**	**Pagaza**	32	8	21	3	0.3	0	17	15	0.23	2	29	1	0.15
	**Outbreak**	**Larabanga**	24	5	16	3	0.27	13	0	11	0.77	0	24	0	0
		**Total**	**56**	**13**	**37**	**6**		**13**	**17**	**26**		**2**	**53**	**1**	

*Abbreviations*: *VV* wild type (susceptible), *VL* heterozygotes, *LL* mutant (resistant), *VI* heterozygotes, *II* mutant (resistant), *FF* wild type (susceptible), *FC* heterozygotes, *CC* mutant (resistant), *n* sample size

## Discussion

Due to their capacity to spread a variety of arboviral infections like dengue, chikungunya, Zika, and yellow fever, *Aedes* mosquitoes pose an increasing hazard to public health on a global scale [34]. Ghana is endemic for yellow fever with the most recent outbreak occurring in 2021 [13]. Hence, the need to monitor the densities and insecticide susceptibility status of *Aedes* mosquitoes in the country. This study sought to determine the risk of arboviral transmission and insecticide resistance status of *Aedes* mosquitoes in a yellow fever outbreak and non-outbreak areas in Ghana. From the data obtained, car tyres were the most representative breeding sites for *Aedes* mosquitoes seasonally. Moreover, the outbreak area (Larabanga) had a high Stegomyia indices value and hence satisfied the WHO criteria for a high-risk zone for yellow fever transmission. Adult *Aedes aegypti* was the predominant vector sampled in all the study sites, with high abundance in the dry season and from outdoor collection. The distribution of larval habitat types varied significantly between seasons.

Findings from this study showed that car tyres were responsible for over 60% of *Aedes* immatures over the entire sampling period. Car tyres seems to provide an optimal temperature, humidity and sufficient light intensity to ensure larvae development [35]. This finding is consistent with studies of Owusu-Asenso et al*.* [11] in Ghana and Kamgang et al*.* [36] in the Central African Republic who also found car tyres as the most conducive and productive breeding habitat for *Aedes aegypti*.

In this study, results from the larval indices indicate that Larabanga had values that exceeded the WHO threshold and hence is a high-risk zone for arboviral pathogen transmission. This is similar to a previous study done by Appawu et al. [10] in Larabanga, where the Stegomyia indices exceeded the threshold and was considered as a high-risk zone for yellow fever transmission. Furthermore, Stegomyia indices values within the WHO criteria reported in Wenchi, Kpalsogu and Pagaza were sufficient to promote an outbreak within these sites. These findings imply that inhabitants within these study sites are at risk of yellow fever infection, Hence, there is a need to employ preventive measures through vaccination and effective vector control strategies.

Seasonal variation in population density is common seasonally for *Aedes* mosquitoes due to their sensitivity to changes in temperature and rainfall [37]. This study found a significantly higher abundance of *Aedes* immature during the rainy season. The development of mosquitoes, their survival and the effective transmission of pathogens are influenced by humidity, temperature and rainfall [38, 39]. Sufficient humidity and rainfall influence the breeding sites, increasing vector populations [40]. Hence, an increase in breeding sites may explain the observed increase in *Aedes* immatures in the rainy season.

This finding corroborates with studies in Ghana by Owusu-Asenso et al. [11] and in Kenya by Ndenga et al*.* [41]. Their findings showed high densities of *Aedes* immatures in the rainy season. However, this finding was contrary to another study in Ghana where *Aedes* larvae were found predominantly in the dry season. He reported that during drought conditions, the surge in the storage of water creates more breeding habitats for *Aedes* mosquitoes, causing an increase in their abundance [10].

It was observed that adult *Aedes* mosquitoes were predominantly collected during the dry season and in outdoor settings. These study areas lacked boreholes and piped water, residents tend to store water in artificial containers, providing persistent breeding sites for *Aedes* mosquitoes even in the dry season. Furthermore, warmer temperatures in the dry seasons enhance the growth of mosquitoes [37]. The life-limiting elements of latitude, altitude, temperature, rainfall, humidity, season, habitat, and dispersal have an impact on the distribution and population of *Aedes* mosquitoes [10]. Finding more adult *Aedes* mosquitoes outside may suggest their exophilic nature as reported in previous studies in Ghana and Kenya [11, 42]. It is important to note that people spending more time outdoors compared to indoors influences the biting and feeding behaviour of *Aedes* mosquitoes [41]. Studies in Ghana have also suggested that *Aedes* mosquitoes often rest outdoors before and after blood feeding [43] and were more abundant from outdoor collection in the dry season [10].

It was observed in this study that the majority of the adult *Aedes* mosquitoes were *Aedes aegypti* which is responsible for yellow fever transmission in Ghana and can transmit other arboviral pathogens such as dengue fever virus [12]. There have been previous reports of dengue viral infections in children in Ghana [14] and exposure to dengue and chikungunya [15, 16, 44] that show the role of *Aedes aegypti* in the transmission of multiple arboviral pathogens in Ghana, which cannot be overlooked.

In this study, *Aedes* mosquitoes across the sites showed resistance to deltamethrin. This might be due to the indirect impact of the use of insecticides for public health vector control such as the use of Long-Lasting Insecticidal Nets (LLINs) and IRS, as well as pesticide use in agriculture [11, 45, 46]. This finding is similar to that reported in a study conducted in Ghana [9]. Whereas *Aedes* mosquitoes collected from Pagaza and Kpalsogu showed suspected resistance to Permethrin, samples from Larabanga were found to be susceptible to Permethrin. Similarly, pyrethroid resistance has also been reported in *Aedes aegypti* populations from Ghana and other West African countries [11, 17, 18, 31] *Aedes* mosquitoes in this study were also resistant or possibly resistant to Pirimiphos-methyl. The findings suggest that resistance to this organophosphate by the *Aedes* population has increased. The observed resistance or possible resistance may be due to intensive and prolonged use of pesticides in agricultural and insecticides in public health including the use of aerosol sprays and coils and IRS in Kpalsogu exerts strong selective pressure, favouring resistant individuals. Cross-resistance may occur due to exposure to other insecticides with similar modes of action and genetic adaptations, such as mutations in acetylcholinesterase or overexpression of detoxification enzymes, which enhance the mosquitoes' ability to metabolize and detoxify the insecticide [47].

In this study, the F1534C and V1016I kdr mutations were high in frequencies in resistant and susceptible *Aedes* mosquitoes while V410L kdr mutation showed low frequencies. Although suspected resistance and susceptibility to permethrin were recorded in the *Aedes* mosquitoes from the same population, there is the risk of resistance developing over time due to the high frequencies of the F1534C and V1016I kdr mutations. Pyrethroid resistance in *Aedes aegypti* is a worldwide challenge for mosquito control due to its use for insecticide-treated nets and indoor residual spraying [47]. Similarly, other studies in Ghana have found high frequencies of V1016I and F1534C kdr mutations in both pyrethroid-susceptible and resistant *Aedes* mosquitoes collected in Ghana [9, 21]. This suggests that other resistance mechanism such as metabolic resistance may be involved in insecticide resistance of *Aedes aegypti* populations in Ghana. Hence, further studies are needed to elucidate the mechanisms mediating insecticide resistance in *Aedes* mosquitoes in Ghana.

## Conclusion

Our findings revealed that *Aedes* immatures were most abundant in the dry season and car tyres were the most representative habitat type. *Aedes aegypti* were the predominant species found. The results showed that Larabanga, the epicentre of the yellow fever outbreak is a high-risk zone for arboviral pathogen transmission. In addition, high phenotypic and genotypic resistance was observed in *Aedes* mosquito populations in Ghana. Surveillance of *Aedes* mosquito bionomics and insecticide susceptibility in Ghana is crucial to help in the development of arboviral vector control strategies to control and prevent arboviral outbreaks in Ghana.

## Data Availability

All the data supporting this study are included in the article.

## Abbreviations

*Ae*.: *Aedes*
DNA: Deoxyribonucleic acid
PCR: Polymerase chain reaction
PMI: President Malaria Initiative
WHO: World Health Organization
YF: Yellow Fever
HLC: Human Landing Catch
PPK: Prokoopack
BG: Biogent sentinel trap
GHS: Ghana Health Service
HI: House Index
CI: Container Index
BI: Breateau Index
VHF: Viral Haemorrhagic Fever
GPS: Geographical position system
